# A Defect Detection Model for Industrial Products Based on Attention and Knowledge Distillation

**DOI:** 10.1155/2022/6174255

**Published:** 2022-10-10

**Authors:** Ze-Kai Zhang, Ming-Le Zhou, Rui Shao, Min Li, Gang Li

**Affiliations:** Shandong Computer Science Center, Qilu University of Technology (Shandong Academy of Sciences), Jinan 250013, China

## Abstract

Industrial quality detection is one of the important fields in machine vision. Big data analysis, the Internet of Things, edge computing, and other technologies are widely used in industrial quality detection. Studying an industrial detection algorithm that can be organically combined with the Internet of Things and edge computing is imminent. Deep learning methods in industrial quality detection have been widely proposed recently. However, due to the particularity of industrial scenarios, the existing deep learning-based general object detection methods have shortcomings in industrial applications. This study designs two isomorphic industrial detection models to solve these problems: T-model and S-model. Both proposed models combine swin-transformer with convolution in the backbone and design a residual fusion path. In the neck, this study designs a dual attention module to improve feature fusion. Second, this study presents a knowledge distiller based on the dual attention module to improve the detection accuracy of the lightweight S-model. According to the analysis of the experimental results on four public industrial defect detection datasets, the model in this study is more advantageous in industrial defect detection.

## 1. Introduction

Quality detection is an important task in the industrial production process, which is of great significance to protect the personal safety of users and avoid economic losses. In industrial quality detection, big data analysis technology, the Internet of Things, edge computing, and other technologies are widely used. Studying an industrial detection algorithm that can be organically combined with the Internet of Things and edge computing is imminent. Early machine vision algorithms used manual feature selection and trained classifiers to identify defect features. This approach relies too much on the robustness of the extracted features, resulting in much time-consuming development.

**Figure 1:**
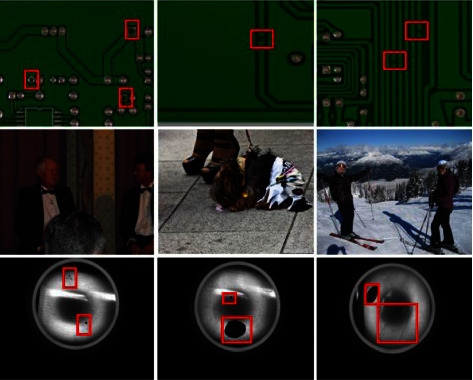
Comparison of industrial detection dataset with COCO dataset. The first row is the picture of the PCB defect dataset. The second row is the COCO dataset pictures. The third row is a picture of aluminium defects. It can be seen that in the field of industrial quality detection, there are many types of small objects, and the positive and negative samples are not balanced, making detection difficult.

In recent years, convolutional neural networks (CNNs) have rapidly developed in image classification, object detection, and image segmentation. However, due to the particularity of industrial defect detection scenarios, deep learning has not been applied widely. As shown in Figure 1, the brightness, colour, object size, and background discrimination of industrial pictures differ from natural scenes. Specifically, the industrial surface defect detection datasets scale is relatively small, unlike large-scale general datasets such as ImageNet, PASCAL VOC2007/2012, and COCO in classical computer vision tasks. Moreover, the industrial datasets differ significantly from the public datasets in the number of samples, sample scale, and proportion of positive and negative samples. This difference leads to less application of general-purpose object detectors in industrial product surface defect detection. In addition, most industrial detection scenarios are offline and require a light model, while existing general object detection models are challenging to meet actual needs.

This study proposes a novel surface defect detection model for industrial products. The model consists of two submodels, T-model and S-model. T-model has a large depth and high detection accuracy and is suitable for scenarios with no obvious requirements for speed. S-model has a small depth and high speed and is more suitable for edge computing scenarios with high-speed requirements. Both models consist of a backbone, neck, and detector. In the backbone, this study combines CNN and swin-transformer. CNN extracts local information in shallow features, swin-transformer extracts global information in deep layers, and each layer outputs weighted features through a unified residual path. In neck, this study designs a dual attention structure to focus on the features of object regions. In detector, this study designs multiple predictions heads on T-model and S-model.

In order to improve the detection effect of the lightweight S-model, a knowledge distiller is presented in this study. It contains the foreground attention-guided distillation of objects and global distillation. The knowledge distiller can significantly improve the detection accuracy of the S-model without extra overhead.

Overall, this study organically combines some existing techniques to create two detection models. An attention module is independently designed inside the model, and a knowledge distiller is designed to transfer the teacher network's knowledge for the lightweight network's guided training. The detection model designed in this study can not only be used for defect detection but also has a wide range of application scenarios in the direction of processing a large amount of image data in the cloud platform combined with the Internet of Things.

The main contributions of this study are listed as follows:

1. This study proposes a novel model for surface defect detection of industrial products. Compared with the traditional CNN-based target detection methods, the model combines CNN and swin-transformer, which significantly improves the accuracy and can be used for application deployment in many image data analyses, edge computing, and other scenarios.
2. This study designs two isomorphic object detection models. In backbone, this study extracts local and global important features by combining CNN and swin-transformer and designs a unified residual path to fuse features at different levels. In the neck, the attention module is designed to improve the effect of feature fusion. At the same time, this study designs the attention module to the decoupling detection head to improve detection accuracy.
3. This study designs a novel attention-guided distillation strategy. The distiller uses the dual attention module to guide the generation of attention region features. The distiller transfers the knowledge of the T-model into the S-model, instructing the S-model to learn the T-model. The distiller in this study can obtain a lightweight, high-accuracy, and fast industrial quality detection model.

## 2. Related Work

### 2.1. Object Detection

In recent years, CNN-based object detection algorithms have been used in many domains. CNN-based object detection algorithms are generally divided into two categories: one stage and two stage. One-stage algorithms include YOLO [1–6], SSD [7], RetinaNet [8], and DSSD[9]. The methods are to directly divide the input image into multiple 1 × 1 grids, where each cell is responsible for detecting objects whose centre points fall within the grid. The methods significantly improve the detection speed, but the accuracy is slow. The two-stage algorithms include Rcnn [10], FastRcnn [11], FasterRcnn [12], and MaskRcnn [13]. These detection algorithms generate boxes via RPN, and the second-level detector uses boxes to conclude. The methods are better at detecting, but the speed is very low.

The one-stage object detector usually contains three parts: backbone, neck, and detector. The backbone is generally composed of multiple groups of convolutions for feature extraction. The well-known backbones include ResNet [14], ResNext [15], VGG [16], DenseNet [17], MobileNet [18], CSPDarkNet [19], and EfficentNet [20], etc. The neck fuses feature maps at different levels in the backbone to enhance the semantic and fine-grained features. Typical structures of neck mainly include FPN [21], PANet [22], Bi-FPN [20], etc. Two types of detectors commonly used in one-stage object detection are as follows: coupled and decoupled.

### 2.2. Attention

The attention mechanism is to make the network pay more attention to the area of the object and ignore the unimportant areas. Its essence is to use the relevant features to learn the weight distribution and then apply the learned weight to the original features. Attention makes the network pay more attention to the target object. Classical attention networks include SENet [23], SKNet [24], ResNext [15], CBAM [25], and self-attention [26].

### 2.3. Vision-Transformer

The transformer [26] is an attention-based encoder-decoder architecture in deep learning. Compared with CNN, vision transformer (Vit) [27] can obtain more refined global attention features and achieve good performance on multiple benchmarks such as ImageNet, COCO, and ADE20k. However, it also has some drawbacks. First, the range of object scales for visual detection varies greatly, and the performance of the Vit [27] may not be optimal in different scenarios. Second, if the image resolution is high, transformer [26] requires much computation. Swin-transformer [28] solves this problem by shifting window partitions to calculate self-attention.

### 2.4. Knowledge Distillation

Knowledge distillation is a widely used method for model compression. Knowledge distillation is to transfer the knowledge of the T-model into S-model to improve the accuracy of lightweight models without adding extra computation. Knowledge distillation methods can be roughly categorized into response based [29–32], feature based [33, 34], and relation based [35]. Response-based methods use the output of the last layer of the teacher network to imitate the teacher's final prediction. This method is simple and efficient, but it relies on the output of the last layer and cannot make the student model obtain the supervision of the middle layer of the teacher model. Feature-based methods use the features in the middle layer of the teacher model to guide the student network to perform feature selection. However, two drawbacks need to be solved urgently. One is choosing the intermediate layer, and the other is matching the feature representation between the intermediate layer and the guiding layer if the layers' sizes are different. Relation-based methods take advantage of the inner product of the features between the two layers and employ the teacher structure as knowledge to guide the student model.

### 2.5. Application in Big Data, Industrial Internet of Things, Defect Detection, and Other Scenarios

Wang et al. proposed a deep learning model [36] combining GRU and LSTM and modeled the crack width of the dam, which can effectively predict the change of dam defects. Chen et al. proposed a training method for CNN and proposed a two-layer parallel training (BPT-CNN) architecture [37] in a distributed computing environment. BPT-CNN effectively improves the training performance of CNN, saves training time while maintaining accuracy, and has wide application fields.

In addition, there are many excellent survey proposed in the fields of big data, industrial Internet of things, etc. Pu et al. proposed an automatic fetal ultrasound standard plane recognition (FUSPR) [38] based on deep learning in an Industrial Internet of Things (IIoT) Environment. Cao et al. proposed a novel BERT-based deep space network (BDSTN) [39] to learn the demand pattern of taxis. Chen et al. combined CNN and LSTM to propose multiple closed spatiotemporal CNNs (MGSTC) [40] for traffic flow prediction; Wang et al. [41] proposed the application of big data technology to data mining, data analysis, and data sharing in large amounts of data, and to create huge economic benefits by using the potential value of data. Zhang et al. [41] summarized the existing blockchain-based systems and applications, which have broad application prospects in different data processing and transmission scenarios. Zhang et al. proposed a combined method of Weber local descriptor (WLD) and local binary pattern (LBP) for seam carving forgery detection [42]. To sum up, some detection methods can be combined with existing technologies such as CNN, not only in defect detection scenarios, but also in different application scenarios such as IoT and big data analysis.

## 3. Method

**Figure 2:**
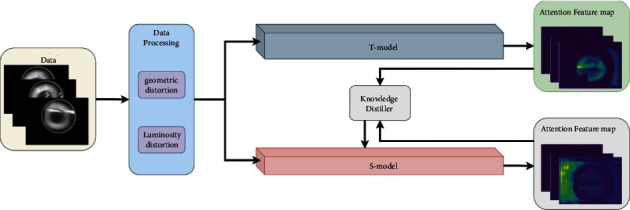
The flowchart of the model in this study. First, the dataset is expanded and enhanced by geometric and photometric transformations. Then, the data is input into the pretrained T-model and S-model, respectively, and the T-model output features guide the S-model training.

The flowchart of the proposed model is shown in Figure 2. The model consists of two submodels: T-model and S-model. In order to improve the detection effect of the lightweight S-model, this study designs a knowledge distiller and a dual attention module. By generating attention features from the pretrained T-model, the S-model training process is guided to learn the features from the T-model. In this way, the S-model reaches or even exceeds the detection effect of the T-model.

**Figure 3:**
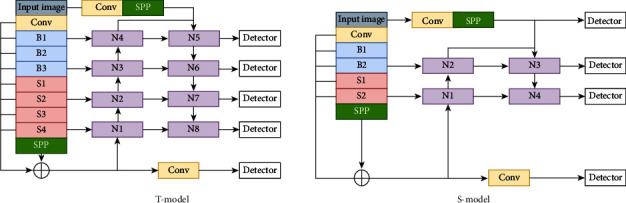
T-model and S-model. The two models are similar in structure, and the depth of the S-model network is much smaller than the T-model. The blue block-B is the Conv layer, the red block-S is the swin-transformer layer, the purple block-N is the feature fusion layer with the dual attention module added, and the detector is the decoupling detection head.

The structure of the T-model and S-model is shown in Figure 3. Both models propose a novel backbone combining convolutional layers with swin-transformer [28] to extract finer-grained image features. This study also adds weighted residual connection paths in the backbone. In the neck, a double-tower structure and dual attention modules are designed to improve the effect of feature fusion. Finally, multiple decoupled detectors are used to detect objects of different scales.

The main formula symbol table used in the rest of the method of this study is annotated as Table 1.

### 3.1. S-Model and T-Model

#### 3.1.1. Backbone

Most general-purpose object detectors are based on CNN and employ large-scale convolution kernels in the first layer of the backbone to increase the receptive field. However, more detailed information will be lost with the increase of convolutional layers. Swin-transformer [28] uses window self-attention to significantly reduce computation load and extract small-scaled features. Therefore, this study designs a novel backbone based on swin-transformer [28].

**Figure 4:**
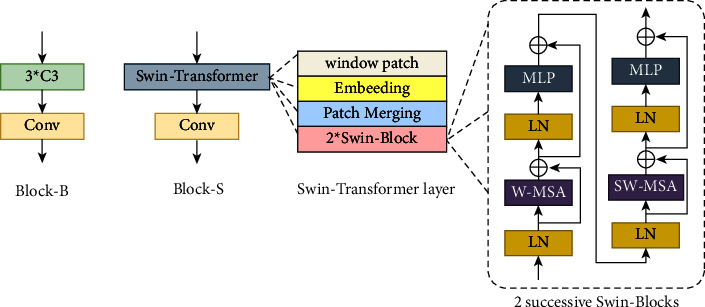
Structure of block-B and block-S.

As shown in Figure 3, our backbone includes block-B, block-S, SPP, and weighted residual fusion paths. The first layer selects a 3×3 convolution kernel to extract fine-scaled features. Block-B comprises three YOLOv5 C3, and three Conv cascaded. Block-S consists of swin-transformer [28], Conv cascade. The structure of the swin-transformer [28] is shown in Figure 4. In swin-transformer [28], the input features are divided into windows and encoded with relative position. The final output is obtained through multiple images down sampling (patch merging) and swin-block. In the steel surface classification experiment of swin-transformer [28], the effect of swin-transformer [28] with relative position encoding is better than that of swin-transformer [28] without it. Therefore, this study adds relative position encoding to block-S.

In order to further improve the detection of small objects, this study designs a unified weighted residual path (Figure 3) and performs fusion of features from the backbone. The fusion formula is as follows:

(1) $$\begin{array}{c} F=f\left(\overset{n}{\underset{i=1}{\sum }}Conv\left(conv\left(x_{i}\right)\right)\frac{{expw}_{i}}{\sum_{1}^{n}{expw}_{j}}\right), \end{array}$$

where *x*_*i*_ is the original input feature and w is the adaptive learnable weight, ∑_1_^*n*^*w*_*j*_=1. *x*_*i*_convolves with a 3×3 kernel to adjust the feature size, a 1×1 to integrate the channel, and then multiplies *expw*_*i*_/∑_1_^*n*^*expw*_*i*_ before fusion.

#### 3.1.2. Neck

**Figure 5:**
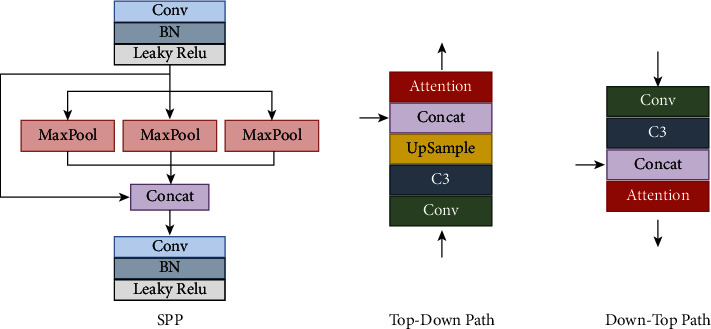
Block-N structure in neck, and block-N is reversed in the top-down and bottom-up paths.

The neck is designed to use better the features extracted by the backbone. In this study, the SPP structure is designed to enhance the invariance and robustness of image features.  Figure 5 shows the structure of block-N in the top-down path and the bottom-up path in the neck. In block-N, this study designs a dual attention module, which will be introduced in detail in Section 3.2.

#### 3.1.3. Decoupled Detector

In object detection, the role of the head in CNN is to regress the generated features to the bounding box and classified into some categories. Most methods use one head for classification and regression. This solution has limitations because classification relies more on fine-grained features, while regression relies more on semantic information to locate the object.

**Figure 6:**
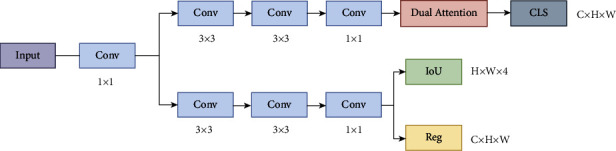
Decoupled detector.

To stress the issue, YOLOX proposes the decoupled detector, which divides classification and regression into two types of problems. In this study, the decoupled detector is innovated (Figure 6), and a dual attention module is added to the classification branch. In our proposal, 6 decoupled detectors are used to detect features of the T-model, and four decoupled detectors to detect features of the S-model. The dense stacking of multiple decoupled detectors helps the models to detect objects of different scales.

### 3.2. Attention Module

Attention is usually embedded in CNNs and used to generate attention matrices to optimize features. CBAM [25] is a classic attention module that combines channel and spatial attention. Specifically, a 1 × 1 × C feature map will be obtained by pooling in channel attention, and a *H* × *W* × 1 feature map will be obtained by pooling in spatial attention. However, pooling leads to much information being lost. A mutual mapping between the three dimensions in a *C* × *H* × W image is crucial to extracting attention.

**Figure 7:**
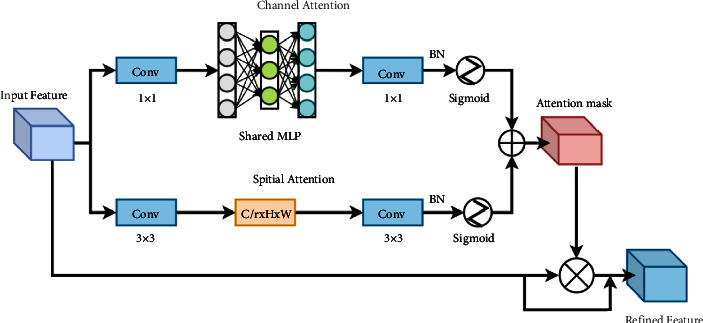
Attention flowchart. This study integrates spatial and channel attention and adds residual paths to them.

The attention structure proposed in this study (Figure 7) includes channel and spatial attention. The resulting attention features are multiplied by input features as their features are combined. In terms of channel attention, this study first uses 1 × 1 convolution instead of pooling to achieve channel interaction and information integration. It then inputs the features into a two-layer neural network (MLP) to amplify the relationship between channels and spatial dimensions. The number of neurons in the first layer in MLP is C/*r* (*r* is the reduction ratio, *r* = 16), the activation function is Relu, and the number of neurons in the second layer is C. In this study, the attention structure introduces BN to reduce the gradient dispersion and speed up the convergence speed. This study uses two dilated convolutions (*d* = 4, *r* = 16) identical to BAM [43] for spatial information integration to focus on spatial information.

The attention extraction formula in this study is as follows:

(2) $$\begin{array}{c} M_{c}\left(F\right)=sigmoid\left(BN\left(Conv\left(MLP\left(Conv\left(F\right)\right)\right)\right)\right), \end{array}$$

(3) $$\begin{array}{c} M_{S}\left(F\right)=sigmoid\left(BN\left(Conv\left(Conv\left(F\right)\right)\right)\right), \end{array}$$

(4) $$\begin{array}{c} F^{\prime }=\left(M_{c}\left(F\right)+M_{s}\left(F\right)\right)⊗F, \end{array}$$

where *M*_*c*_(*F*) denotes channel attention, *M*_*s*_(*F*) denotes spatial attention, and *F*′ denotes the superposition of channel attention and spatial attention and multiplied by the original input *F*.

### 3.3. Knowledge Distillation Module

The lightweight model has speed and memory consumption advantages, but the detection effect is challenging to meet the requirements. Knowledge distillation is an effective method to improve the detection accuracy of small models. The general knowledge distiller focuses on the extraction of the overall features. The formula is as follows:

(5) $$\begin{array}{c} L_{D}=\frac{1}{CHW}\overset{C}{\underset{i=1}{\sum }}\overset{H}{\underset{j=1}{\sum }}\overset{W}{\underset{k=1}{\sum }}{\left|F_{i,j,k}^{T}-f\left(F_{i,j,k}^{S}\right)\right|}^{2}, \end{array}$$

where *FT*/*i*, *j*, *k* and *FS*/*i*, *j*, *k* denote the characteristics of teachers and students, respectively, and *f*() denotes converting *FS*/*i*, *j*, *k* to the same data dimension as *FT*/*i*, *j*, *k*. *H*, *W* specify the height and width of the feature, and *C* denotes the channel.

This study uses knowledge distillation to improve the S-model's performance. The image's foreground and background are distilled separately. The positive and negative samples are separated by separating the foreground and background, which solves the imbalance of positive and negative samples in the image. This study also designs an attention module to generate attention regions, forcing the student model to learn the vital features of the image.

In the distillation method of foreground and background, this study first sets the binary mask of foreground segmentation (the real position of the object frame) and sets objects within the ground truth box to 1 and objects outside the ground truth box to 0. It is expressed as follows:

(6) $$\begin{array}{c} {Mask}_{x,y}=\left\{\begin{array}{cc} 1, & \left(x,y\in GT\right),\\ 0, & otherwise, \end{array}\right. \end{array}$$

where *x* and *y* denote the horizontal and vertical coordinates of the area object and GT denotes the area position of the real frame. When the position of *x*, *y* falls in the real frame area, it is set to 1; otherwise, it is 0.

Due to the large-scale variation of objects in industrial detection datasets and the uneven distribution of positive and negative samples of objects, these will adversely affect the distillation effect. For this reason, this study uses the scaling mask to balance the object scale with reference to FGD [44] to solve the problem of an unbalanced object scale. The formula is as follows:

(7) $$\begin{array}{c} {ScaleMask}_{x,y}=\left\{\begin{array}{c} \frac{1}{H_{x,y}\times W_{x,y}},\left(x,y\in GT\right),\\ \frac{1}{\sum_{x=1}^{H}\sum_{y=1}^{W}\left(1-{Mask}_{x,y}\right)}, \end{array}\right. \end{array}$$

where *GT* is the region of the ground truth. In this study, the scaling mask is used to normalize the foreground and background pixels. When there are two objects in an image, the bounding box of the large object may cover small object. In this study, when small objects and large objects are in a bounding box, the following formula is used:

(8) $$\begin{array}{c} Mask=\min\;\left(GT\right), \end{array}$$

where *GT* represents the real box of the object. When a small object is surrounded by the box of a large object, the smallest bounding box is preferentially selected.

After separating the foreground and background, this study uses the designed dual attention module to generate the attention mask. The attention mask formula is as follows:

(9) $$\begin{array}{c} M\left(F\right)=\left(M_{c}\left(F\right)+M_{s}\left(F\right)\right)⊗F, \end{array}$$

(10) $$\begin{array}{c} AttentionMask\left(F\right)=softmax\left(\frac{M\left(F\right)}{T}\right), \end{array}$$

where *M*(*F*) denotes the process of generating attention, *F* denotes the original feature map, *M*_*c*_(*F*) denotes channel attention, *M*_*s*_(*F*) denotes spatial attention, *T* denotes distillation temperature, *T* = 20, and softmax is used to process features graph weights.

In the distillation process of foreground and background, binary mask, scale mask, and attention mask are used for attention-guided distillation. The loss function is as follows:

(11) $$\begin{array}{c} L_{fg}=\overset{C}{\underset{x=1}{\sum }}\overset{H}{\underset{y=1}{\sum }}\overset{W}{\underset{z=1}{\sum }}{Mask}_{y,z}{ScaleMask}_{y,z}AttentionMask{\left|F_{x,y,z}^{T}-f\left(F_{x,y,z}^{S}\right)\right|}^{2}+\overset{C}{\underset{x=1}{\sum }}\overset{H}{\underset{y=1}{\sum }}\overset{W}{\underset{z=1}{\sum }}{\left({1-Mask}_{x,y}\right)ScaleMask}_{x,y}AttentionMask{\left|F_{x,y,z}^{T}-f\left(F_{x,y,z}^{S}\right)\right|}^{2}, \end{array}$$

where *L*_*fg*_, *Mask*_*y*,*z*_,(1 − *Mask*_*y*,*z*_), *ScaleMask*_*y*,*z*_, *AttentionMask*are the distillation loss of foreground and background, foreground mask, background mask, scale mask, attention mask, respectively, and |*F*_*x*,*y*,*z*_^*T*^ − *f*(*F*_*x*,*y*,*z*_^*S*^)|^2^ is the difference between teacher feature and student feature.

In addition, this study uses the attention loss function to let the student model learn the attention mask of the teacher model. The loss formula is as follows:

(12) $$\begin{array}{c} L_{A}=SmoothL1\left({AttentionMask}^{T},{AttentionMask}^{S}\right), \end{array}$$

where *L*_*A*_ is the attention mask loss function, *AttentionMask*^*T*^ denotes the teacher's attention mask, and *AttentionMask*^*S*^ denotes the student's attention mask.

In the feature distillation of the foreground, this study uses the designed attention module to distil the entire feature map. The loss formula is as follows:

(13) $$\begin{array}{c} {L_{G}=\left|M\left(F_{x,y,z}^{T}\right)-M\left(f\left(F_{x,y,z}^{S}\right)\right)\right|}^{2}, \end{array}$$

where *L*_*G*_ is the distillation loss function of the entire feature map, *M*(*FT*/*x*, *y*, *z*) is the attention feature map of the teacher model, and *M*(*f*(*FS*/*x*, *y*, *z*)) is the attention feature map of the student model.

### 3.4. Loss Function

In order to solve the unbalanced positive and negative samples in the PCB dataset, this study uses QFocal loss as the classification and confidence losses of the T-model and S-model. The formulas are as follows:

(14) $$\begin{array}{c} QFL\left(\sigma \right)=-\alpha_{t}\times {\left|y-\sigma \right|}^{\beta }\times \left[\left(1-y\right)\log\;\left(1-\sigma \right)+ylog\left(\sigma \right)\right], \end{array}$$

where *y* is the smoothed label in [0, 1] and *σ* is the prediction result. Focal loss introduces two factors *α*_*t*_ and |*y* − *σ*|^*β*^, where *α*_*t*_=*y* × *α*+(1 − *y*) × (1 − *α*) is used to balance positive and negative samples, and |*y* − *σ*|^*β*^ is used to stress difficult detected samples. In addition, this study introduces CIoU Loss as the prediction box regression loss of T-model and S-model. The formula is as follows:

(15) $$\begin{array}{c} L_{CIOU}=1-IOU+R_{CIOU}\left(B_{pd},B_{gt}\right), \end{array}$$

where *R*_*CIOU*_ is the penalty term for the prediction box *B*_*p* *d*_ and the object box *B*_*gt*_. CIoU loss considers the overlapping area, centre point distance, and aspect ratio in the prediction frame regression, which solves the problem of inconsistency between the real frame and the predicted frame during object detection. The normalized distance and penalty term between the centre points of the two bounding boxes are defined as follows:

(16) $$\begin{array}{c} {R_{CIOU}=\frac{\rho^{2}\left(b,b_{gt}\right)}{C^{2}}+\alpha \frac{4}{\pi^{2}}\left(\arctan\frac{w^{gt}}{h^{gt}}-\arctan\frac{w}{h}\right)}^{2}, \end{array}$$

where *b*, *b*_*gt*_ denote the centre points of *B*_*p* *d*_, *B*_*gt*_ respectively, *ρ*(*x*) is the Euclidean distance, and *C* is the diagonal length of the smallest bounding box covering these two boxes. *α* is a positive trade-off parameter. *w*, *h* are the width and height of the prediction box. In the selection of anchors, this study uses K-means to filter the anchors that meet the criteria.

In summary, the total loss function proposed in this study is as follows:

(17) $$\begin{array}{c} Total\;Loss=\alpha L_{fg}+\beta L_{A}+\gamma L_{G}+2\delta QFL+\varepsilon L_{CIOU}, \end{array}$$

where *L*_*fg*_, *L*_*A*_, *L*_*G*_, *QFL*, *L*_*CIOU*_ denote the distillation loss of foreground and background, attention mask loss, distillation loss of the entire feature map, QFocal loss, cIou loss, respectively, *α*, *β*, *γ*, *δ*, *ε* are the weight parameters of the balance loss, respectively. In this study, an adaptive weight updater is designed to adjust the weight adaptively. The formula is as follows:

(18) $$\begin{array}{c} W=\frac{\exp\left(L_{i}\right)}{\sum_{i=1}^{n}\exp\;\left(L_{i}\right)}\times n\times w_{k}, \end{array}$$

where *W* denotes the updated weight parameter, *w*_*k*_ denotes the weight parameter before the update, *L*_*i*_ denotes the value of each loss, and *n* denotes the number of weight parameters. In this study, each weight parameter is set to one before the training starts, and then in each training round, the weight parameter is updated according to the proportion of each loss value in the total loss. In this way, the loss with a large value will get a large weight in the next round of optimization, and the weight with a small value will be further reduced. At the end of the training, all tasks can be optimized almost simultaneously.

## 4. Experiments and Analysis

In this section, experiments are carried out on the PCB defect dataset, the NEU surface defect dataset, and the aluminium defect dataset.

### 4.1. Experimental Environment and Parameter Settings

This article implements the code in the PyTorch framework, version 1.9.0. CUDA version 11.4, cuDNN version 8.0. This study's model training and inference are performed on NVIDIA RTX 6000 × 1 and Intel i9-9900k@5 GHz × 1. Experimental platforms are GPU memory of 24 GB and CPU memory of 32 GB. The IDE used in the experiment is Pycharm 2019 Professional Edition.

In the training process, this study uses YOLOv5 as the baseline to build the T-model, uses the pretraining weights of YOLOv5 in the initial training of the T-model, and saves T-model weights after training. This study uses T-model for pretraining, loads the pretraining weights of the T-model to S-model for training, and uses knowledge distillation during the training process. Unless otherwise specified, the network in this study is trained with Adam for 300 iterations with an initial learning rate of 0.001, and the learning rate is adjusted using cosine annealing. This article uses a weight decay of 0.0001 and a momentum of 0.9. Also, the input image is resized to 640 × 640. The batch of the model in this study is eight during training, the batch is one during inferencing, and TensorRT is not used.

### 4.2. Object Detection and Evaluation Indicators

FPS [45]: in this study, the model inference is carried out under the same equipment conditions. The same size image is used to calculate FPS and evaluate the model's processing speed.

IOU [45]: object detection uses the IOU to calculate the degree of coincidence between the predicted box and the ground-truth box, which further measures the accuracy of detecting the corresponding object in a specific dataset.

mAP [45]: mAP is the sum of the average precision of all categories divided by the number of all categories. mAP@.5 is the model accuracy index when the IOU is 0.5. mAP@.5:.95 is obtained by calculating an mAP every 0.5 from IOU from 0.5 to 0.95 and finally averaging these maps.

### 4.3. Experiments on the Aluminium Defect Dataset

**Figure 8:**
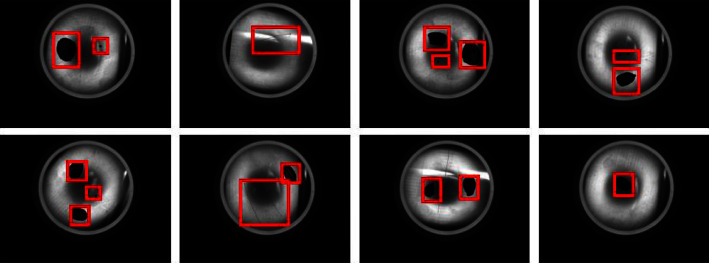
Example of aluminium defect.

The aluminium defect dataset is the images of aluminium surface defects published by Baidu AI (Figure 8). It contains 412 images in total. There are four types of defects, i.e., Zhen_kong, ca_shang, zang_wu, and zhe_zhou, and one image may contain different types of defects. In this study, the dataset is processed with photometric and geometric distortion. Specifically, this study brightens the picture and then flips and pans the picture to expand the dataset, increasing the number of pictures in the dataset to 1236. Then, the extended images are divided into a training set, validation set, and test set with a ratio of 8 : 1:1. It can be seen from Figure 8 that the surface defect scale of aluminium material varies greatly, which brings difficulties to the detection.

#### 4.3.1. Comparative Experiments

Table 2 lists the comparison experiment results of aluminium defect datasets. It can be seen that the T-model of this study surpasses most classic object detection models, indicating that the model is more suitable for the field of industrial detection. T-model size is smaller than YOLOv5X, YOLOv4, YOLOR-P6, Faster-R-CNN, etc. The F1-score and mAP of the T-model are all in the leading position. T-model surpassed the newly proposed YOLOR 1.13% on mAP@.5, and F1-score exceeded 0.05. YOLOX has better detection performance than T-model, but T-model accuracy is close to YOLOX. However, due to the swin-transformer, the FPS advantage of the T-model is not apparent. In contrast, the S-model is lighter, the reasoning speed is fast, and the accuracy after knowledge distillation is close to the T-model.

**Figure 9:**
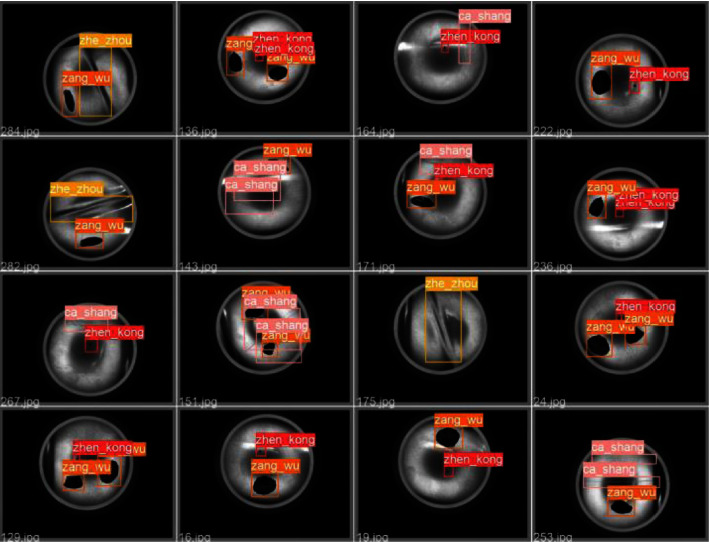
Effect of aluminium defect detection.

In order to more intuitively show the detection effect of the T-model, Figure 9 shows the detection results of 16 pictures.

#### 4.3.2. Ablation Experiments

Table 3 lists the ablation experiments performed with YOLOv5S as the baseline. In the ablation experiment of the backbone, this study uses the B4, S3, and SPP outputs in the backbone as the input of YOLOv5S neck for experimenting. The results in the second row show that when using the backbone of the T-model, mAP@.5 is 0.63% higher than that of YOLOv5S, and the other indicators are also slightly improved. On the neck ablation experiment, this study experiments with three C3s in the YOLOv5S backbone with one SPP output as the neck input. The third row shows that the detection effect is improved when introducing the neck in this study.

This paper also conducts ablation experiments with T-model as the baseline to verify the residual fusion path in the backbone and the down sampling path in neck. The ablation results are listed in Table 4. The first row shows the results without weighted fusion paths in the backbone and down sampling paths in neck. The second row shows that when adding the residual fusion path to the backbone, mAP@.5 increases by 0.58%, and the F1-score increases by 0.02. The third rows show that when introducing the down sampling path in neck, mAP@.5 is improved by 0.52%, and the F1-score is improved by 0.02. In general, the residual fusion path and the down sampling path are beneficial to industrial detection scenarios with drastic changes in scale.

#### 4.3.3. Experiments on the Detector

This study examines several different detectors, including coupled detector, decoupled detector, and decoupled detector with added dual attention module. This study conducts experiments with T-model as the baseline. Specifically, this study experiments with these detectors on T-model, and the results are listed in Table 5. The experimental results in the second row show that the mAP@.5 and F1-score of decoupled detector are improved by 0.78% and 0.05, respectively, compared with coupled detector. The third line shows that the detection accuracy is further improved when adding the dual attention module to the classification branch of decoupled detector, indicating that the dual attention module improves the classification accuracy.

#### 4.3.4. Experiments on the Dual Attention Module

**Figure 10:**
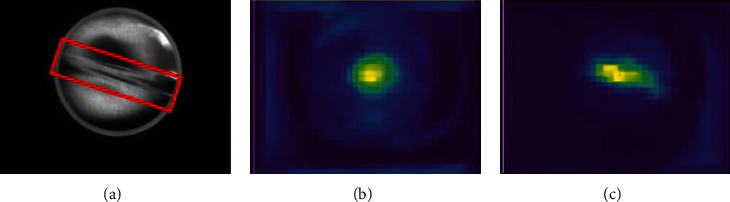
(a) Original image. (b) CBAM heatmap. (c) Dual attention module heatmap. The darker the yellow, the higher the attention.

This study analyses the features of CBAM. As shown in Figure 10, it is a heat map comparison between CBAM and the dual attention module. It can be seen that the dual attention module pays more attention to object areas such as wrinkles.

This study uses T-model as the baseline for experiments on the dual attention module (DA). The experimental results are listed in Table 6. It can be seen that the dual attention module effect surpasses CBAM.

#### 4.3.5. Knowledge Distillation

This study conducts experiments related to knowledge distillation on lightweight models. The experiments are performed on isomorphism object detectors and heterogeneous object detectors, respectively. On isomorphic object detectors, this study experiments T-model and S-model, Efficientdet-d7, and Efficientdet-d3. This study experiments with faster-R-CNN-ResNet50, faster-R-CNN-VGG16, YOLOv4, and YOLOv4-tiny on heterogeneous object detectors. In addition, this study also experimented with output layer knowledge distillation on T-model and S-model as a comparative experiment.  Table 7 lists the experimental results of various classic object detectors. It can be seen that knowledge distillation improves the detection performance of the S-model, especially when T-model and S-model are isomorphic. It proves that the knowledge distiller can improve the detection accuracy of small models without increasing the number of parameters.

### 4.4. Experiments on the PCB Defect Dataset

**Figure 11:**
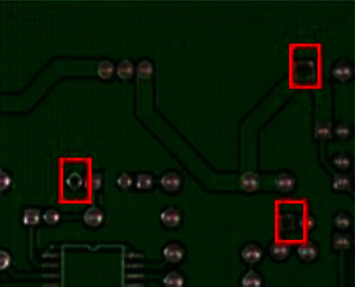
Missing hole.

The PCB defect dataset has 1386 images, and its annotation files contain the object location information and classification. It contains six defects: missing hole, mouse bite, open circuit, spur, short, and spurious copper. Each image may contain multiple defective objects of the same type (missing holes in Figure 11). In the experiments in this study, 900 images of different defect types are selected for training (mainly minor defects that are difficult to identify). In our experiment, images and annotations are divided into the training set, validation set, and experiment set according to the ratio of 6 : 2 : 2.

This study conducts a comparative experiment on the PCB surface defect dataset, omitting FPS since the input size is still 640. It should be noted that the effect of the S-model is the result of distillation through the knowledge distiller. The comparison experiments are listed in Table 8. It can be seen that most of the classic object detection models are not effective for small object detection, and the T-model in this study shows a better performance.

**Figure 12:**
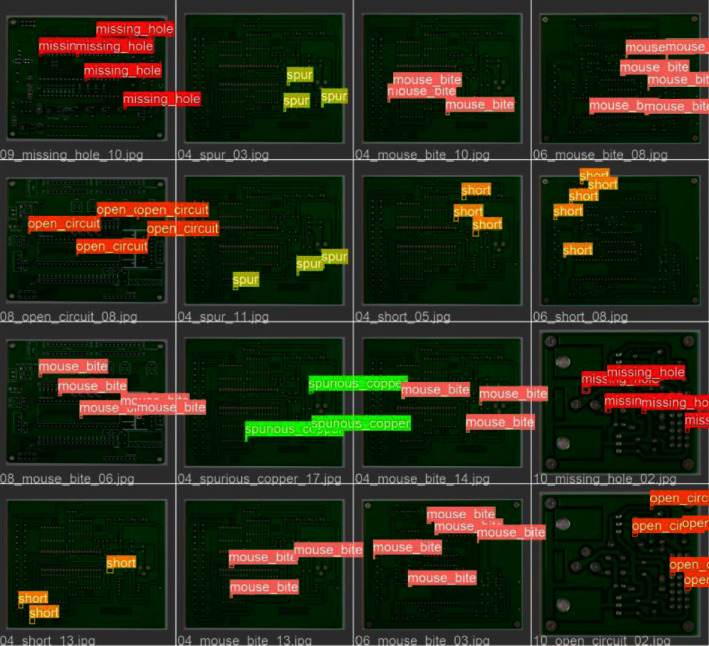
PCB defect dataset detection results.

In order to more intuitively show the detection effect of the T-model, Figure 12 shows the detection results of 16 pictures.

#### 4.4.1. Ablation Experiment

Table 9 lists the ablation experiments performed in this study with YOLOv5S as the baseline. The experimental operations are consistent with the experiments on the aluminium defect dataset. The results in the second row show that after using the backbone of the T-model in this study, mAP@.5 is 3.68% higher than that of YOLOv5S indicating that the backbone in this study is beneficial for small object detection. The third line shows that the detection effect is improved when introducing the neck of the T-model. The dual attention module makes the network pay more attention to the object and improves the small object detection effect.

This study also uses T-model as the baseline to conduct ablation experiments to verify the residual fusion path in the backbone and the down sampling path in the neck. The experimental operations are consistent with the experiments on the aluminium defect dataset. The ablation test results are listed in Table 10. The first row shows the results without weighted fusion paths in the backbone and down sampling paths in neck. The second row shows that when adding the residual fusion path to the backbone, the effect is significantly improved, indicating that the residual path can better fuse the fine-grained features in the backbone. The third row shows that by introducing the down sampling path in neck, mAP@.5 is improved by 2.4%, and the F1-score is improved by 0.03. It shows that the down sampling path is beneficial for small object detection because more fine-grained feature information is fused into neck. Overall, the residual fusion and the down sampling paths can improve the detection effect.

### 4.5. Experiments on the NEU Surface Defect Dataset

North-eastern university releases the NEU surface defect dataset. This dataset collects six typical defects on the surface of the hot-rolled strip: rolled-in scale, patches, crazing, pitted surface, inclusion, and scratches. Each image has several defects of the same type. The label file marks the category and specific location of the defective object.

**Figure 13:**
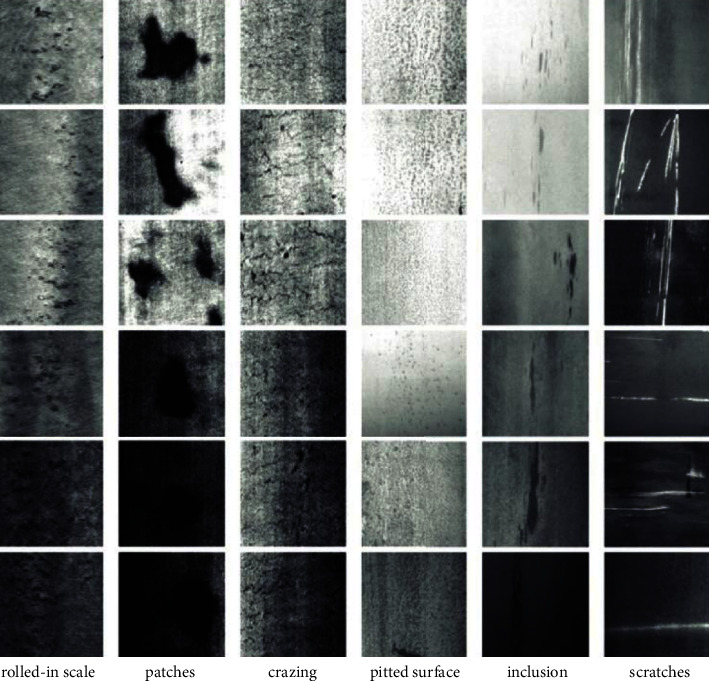
Example of surface defects in steel.

Figure 13 shows different kinds of steel defects. The categories' defects have significant differences in appearance. Such scratches (last column) could be horizontal, vertical, slanted, etc. At the same time, each category has similarities in defects, such as rolled-in scale, crazing, and pitted surface. In addition, due to the influence of lighting and specific materials, the grey value of each category of defect images will also change. Object detection in the NEU surface defect dataset contains three difficulties: intraclass defects have significant appearance differences, interclass defects have similar aspects, and defect images are affected by changes in lighting and specific materials. According to the observation of the dataset, some cracks are concentrated in one direction because the steel is slender, and scratches are in any direction. Cracks and scratches are very similar, so scratches in specific directions are easily identified as cracks. Therefore, this study increases the data enhancement of cracks, rotates, splices crack pictures, and reduces the recognition error rate.

**Figure 14:**
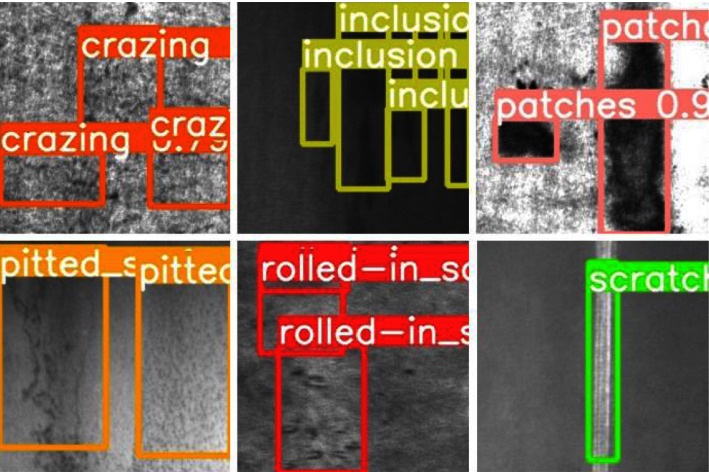
Steel defect detection effect.

In order to show the generalization ability of this model in industrial detection, this study conducts a comparative experiment on the NEU surface defect database, where the S-model is the result of knowledge distillation. The comparative test results are listed in Table 11. It can be seen that the T-model and S-model still have better performance, indicating that the object detection model of this study has a particular generalization ability in multiple industrial detection datasets.  Figure 14 shows the different steel defect recognition results; it can be seen that all the defects are accurately identified.

### 4.6. Experiments on Glass Bottle Bottom Mould Point Dataset

This is a dataset for recognising mould point sequences at the bottom of glass bottles, with different permutations representing different product lot numbers. The mould point identification on the bottom of glass bottles is mainly used to locate the batch of glass products online to trace the product. The dataset contains 900 glass bottle bottom mould point images, each with a native resolution of 800 × 780 and 18 types. Each image has a corresponding label file. In this study, the label files correspond to the images one by one, and the training set, test set, and validation set are divided according to the ratio of 6 : 2 : 2.

**Figure 15:**
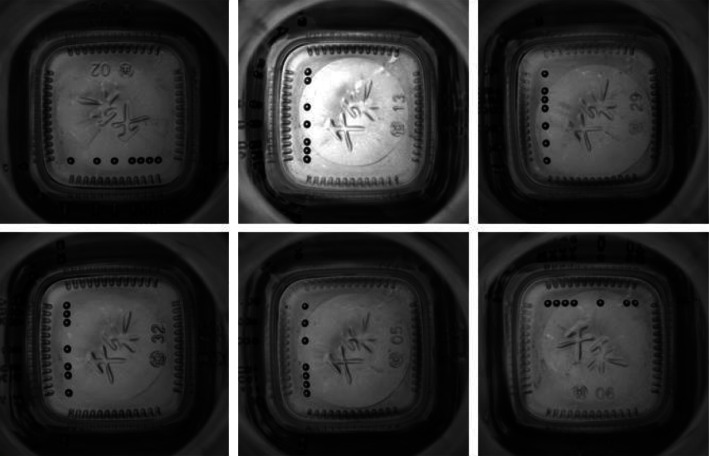
Example of bottom mould point data.

**Figure 16:**
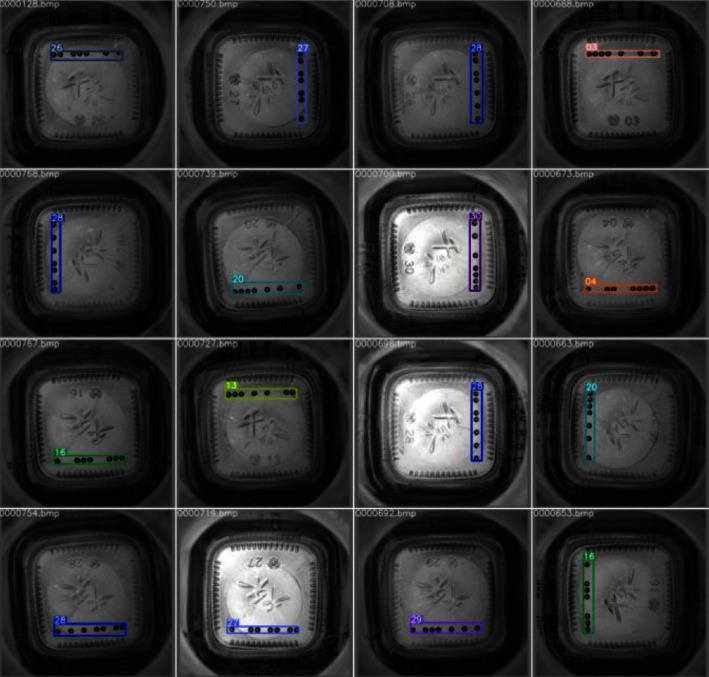
Glass bottle bottom mould point recognition effect.

Figure 15 shows the images of different glass bottle bottom mould points, and the high similarity between objects makes detection difficult. In order to demonstrate the generalization ability of the model in industrial detection, this study conducts a comparative experiment on the glass bottle bottom mould point database, where the S-model results from knowledge distillation. The comparison test results are listed in Table 12. It can be seen that T-model and S-model still have better performance, indicating that the object detection model in this study has a specific generalization ability on multiple industrial detection datasets.  Figure 16 shows the recognition effect of 16 different model point images. It can be seen that all model point objects are accurately recognized and positioned.

## 5. Conclusions

This study proposes a deep learning model for industrial quality detection. The model consists of T-model and S-model, which aims to meet detection tasks under different conditions. The model uses a combination of swin-transformer and convolution to extract the global information of the image. A dual attention module is designed to improve the neck's attention to important areas of the image, thereby improving the detection effect of the model. This study also designs a knowledge distiller using a dual attention module to improve the detection effect of the S-model. Finally, this study designs an adaptive loss weight updater to adjust the loss weights automatically. The experimental results show that the T-model in this study has high accuracy and is suitable for online data processing in scenarios such as IoT intelligent computing and big data analysis. The S-model in this study is fast and suitable for use in scenarios such as edge computing. In general, the model in this study can meet the needs of different scenarios and achieve a balance between accuracy and speed.

## Figures and Tables

**Table 1:** The main formula symbol used in the rest of the method.

Formula symbol annotation	Location
*x*_*i*_: the original input feature	Formula (1)
*w*_*i*_: the learnable weight of a single feature map	
*w*_*j*_: the weight of any feature map to be accumulated.	

*F*: original input feature	Formulas (2)–(4)
*M*_*c*_(*F*): channel attention	
*M*_*S*_(*F*): spatial attention	
*F*′: the superposition of channel attention and spatial attention and multiplied by the original input *F*.	
⊗: multiply by bit	

*F*_*i*,*j*,*k*_^*T*^: the characteristics of teachers	Formula (5)
*F*_*i*,*j*,*k*_^*S*^: the characteristics of students	
*f*(): converting *F*_*i*,*j*,*k*_^*S*^ to the same data dimension as *F*_*i*,*j*,*k*_^*T*^	
*H*, *W*: specify the height and width of the feature	
*C*: channels	

*x*, *y*: the horizontal and vertical coordinates of the area object	Formula (6)
*GT*: the area position of the real frame	

M(F): the process of generating attention	Formulas (9) and (10)
*F*: the original feature map	
T: distillation temperature, T = 20	

*L*_*fg*_: the distillation loss of foreground and background	Formula (11)
*Mask*_*y*,*z*_: foreground mask	
(1 − *Mask*_*y*,*z*_): background mask	
*ScaleMask*_*y*,*z*_: scale mask	
*AttentionMask*: attention mask	
|*F*_*x*,*y*,*z*_^*T*^ − *f*(*F*_*x*,*y*,*z*_^*S*^)|^2^: the difference between teacher feature and student feature	

*M*(*F*_*x*,*y*,*z*_^*T*^): the attention feature map of the teacher model	Formula (13)
*M*(*f*(*F*_*x*,*y*,*z*_^*S*^)): the attention feature map of the student model	

*L*_*fg*_: the distillation loss of foreground and background	Formula (17)
*L*_*A*_: attention mask loss	
*L*_*G*_: distillation loss of the entire feature map	
*QFL*: QFocal loss	
*L*_*CIOU*_: CIou loss	
*α*, *β*, *γ*, *δ*, *ε*: the weight parameters of the balance loss	

*W*: the updated weight parameter	Formula (18)
*w*_*k*_: the weight parameter before the update	
*L*_*i*_: the value of each loss	
*n*: the number of weight parameters	

**Table 2:** Comparative experiment of the aluminium defect dataset.

Approach	Backbone	mAP@.5 (%)	mAP@.5-.95 (%)	Precision (%)	Recall (%)	F1	Model size (M)	FPS
SSD	VGG-16	86.58	35.2	99.8	62.5	0.77	99.76	67
Faster-rcnn	ResNet-50	92.35	42.3	73.88	97.5	0.84	523	16
YOLOv3	DarkNet-53	95.9	54.4	94.7	95.7	0.95	234	19
YOLOv5S	CSPDarkNet	97.2	56.6	96.4	96.8	0.97	26	50
Efficientdet-d3	EfficientNet-B3	93.52	50.2	91.85	90.91	0.91	14.78	20
YOLOv5X	CSPDarkNet	94.3	54.9	95.1	94.7	0.95	167	21
Centrenet	Hourglass-104	60.3	38.6	51.09	60.61	0.55	124.61	48
Retinanet	ResNet-101	96.52	46.7	94.79	93.32	0.94	144.84	20
YOLOv4	CSPDarkNet-53	94.13	59.1	83.38	96.01	0.89	245.53	29
YOLOR-P6	CSPDarkNet	97	53.1	88.3	97.8	0.93	140.88	61
YOLOX-s	Darknet-53	99.2	58.2	98.91	99.3	0.99	34.2	41
T-model	Our backbone	98.53	58.8	98.85	98.38	0.98	81.6	19
S-model	Our backbone	97.82	55.86	97.83	96.87	0.97	25.1	36

**Table 3:** Ablation experiment with YOLOv5S.

Approach and improvement	mAP@.5 (%)	mAP@.5-.95 (%)	Precision (%)	Recall (%)	F1
YOLOv5S	97.2	56.6	96.4	96.8	0.97
+Our backbone	97.83	56.94	97.84	96.87	0.97
+Our neck	97.71	57.13	97.1	97.38	0.98

**Table 4:** Ablation experiment with the T-model.

Approach and improvement	mAP@.5 (%)	mAP@.5-.95 (%)	Precision (%)	Recall (%)	F1
T-model	97.91	57.43	97.24	97.87	0.97
+Residual fusion path	98.02	57.57	98.16	98.47	0.99
+Residual fusion path + down sampling path	98.53	58.8	98.85	98.38	0.98

**Table 5:** Experiments on three detectors.

Detector	mAP@.5 (%)	mAP@.5-.95 (%)	Precision (%)	Recall (%)	F1
Coupled detector	97.24	57.05	86.4	97.31	0.93
Decoupled detector	98.02	57.7	98.16	98.47	0.98
Decoupled detector + attention	98.53	58.8	98.85	98.38	0.98

**Table 6:** Comparison experiment between CBAM and dual attention module.

Approach	mAP@.5 (%)	mAP@.5-.95 (%)	Precision (%)	Recall (%)	F1
T-model without DA	97.52	57.2	96.75	97.87	0.97
+DA	98.53	58.8	98.85	98.38	0.98
+CBAM	96.68	53.15	95.75	96.83	0.96

**Table 7:** Knowledge distiller experiments.

Models	mAP@.5 (%)	mAP@.5-.95 (%)	Precision (%)	Recall (%)
S-model	95.76	54.23	95.82	95.25
S-model with KD	97.82	55.86	97.83	96.87
Faster-rcnn-VGG16	87.22	36.18	77.42	88.84
Faster-rcnn-VGG16 with KD	87.87	36.26	87.46	86.67
Efficientdet-d3	93.52	50.2	91.85	90.91
Efficientdet-d3 with KD	95.8	51.34	91.88	96.78
YOLOv4-tiny	84.43	35.79	80.46	86.01
YOLOv4-tiny with KD	84.94	35.6	80.44	86.9

**Table 8:** Comparative experiment of PCB surface defects.

Approach	Backbone	mAP@.5 (%)	mAP@.5-.95 (%)	Precision (%)	Recall (%)	F1
SSD	VGG-16	17.1	10	19.93	54.64	0.29
Faster-rcnn	ResNet-50	78.33	50.24	67.74	74.51	0.7
YOLOv3	DarkNet-53	73.32	47.50	90.85	52.66	0.67
YOLOv5S	CSPDarkNet-53	91.2	51.8	81.82	92.12	0.86
Efficientdet-d3	EfficicientNet-B3	71.8	35	99.44	49.51	0.66
YOLOv5X	CSPDarkNet-53	91.8	50.7	95.2	91.3	0.93
Centrenet	Hourglass-104	43.4	14.53	43.79	52.2	0.48
Retinanet	ResNet-101	13.18	5	66.67	4.31	0.08
YOLOv4	CSPDarkNet-53	81.2	50.06	89.37	94.78	0.92
YOLOR-P6	CSPDarkNet	94.7	54.5	93.1	93.1	0.94
YOLOX-s	Darknet-53	95.84	62.03	87.16	93.73	0.91
T-model	Our backbone	94.79	58.02	88.14	95.65	0.91
S-model	Our backbone	92.51	56.98	83.79	92.33	0.88

**Table 9:** Ablation experiment with YOLOv5S.

Approach and improvement	mAP@.5 (%)	mAP@.5-.95 (%)	Precision (%)	Recall (%)	F1
YOLOv5S	91.2	51.8	81.82	92.12	0.86
+Our backbone	91.98	52.48	96.05	92.46	0.94
+Our neck	92.05	53.09	96.62	93.6	0.95

**Table 10:** Ablation trials with T-model.

Approach and improvement	mAP@.5 (%)	mAP@.5-.95 (%)	Precision (%)	Recall (%)	F1
T-model	92.39	54.21	91.71	89.84	0.88
+Residual fusion path	93.65	56.44	89.94	94.43	0.92
+Residual fusion path + down sampling path	94.79	58.02	88.14	95.65	0.91

**Table 11:** Comparative experiments on NEU defect datasets.

Approach	Backbone	mAP@.5 (%)	mAP@.5-.95 (%)	Precision (%)	Recall (%)	F1
SSD	VGG-16	66.73	26.6	87.27	34.52	0.5
Faster-rcnn	ResNet-50	76.95	39.9	44.14	87.86	0.59
YOLOv3	DarkNet-53	68.9	33.5	72.2	65.9	0.7
YOLOv5S	CSPDarkNet-53	70.9	35.6	78.4	64.3	0.35
Efficientdet-d3	EfficicientNet-B3	65.65	34	84.76	48.44	0.77
YOLOv5X	CSPDarkNet-53	72	37.7	66.8	72.5	0.69
Centrenet	Hourglass-104	39.24	13.5	55.5	18.2	0.29
Retinanet	ResNet-101	66.54	31.9	81.73	44.62	0.57
YOLOv4	CSPDarkNet-53	68.73	32.9	95.6	40.54	0.56
YOLOR-P6	CSPDarkNet	76.52	38.3	50.86	83.37	0.63
YOLOX-s	Darknet-53	79.38	40.23	53.73	84.88	0.66
T-model	Our backbone	75.56	39.68	53.45	83.16	0.65
S-model	Our backbone	74.41	39.2	49.05	82.28	0.62

**Table 12:** Comparative experiments on NEU defect datasets.

Approach	Backbone	mAP@.5 (%)	mAP@.5-.95 (%)	Precision (%)	Recall (%)	F1
SSD	VGG-16	70.44	32.52	78.94	49.48	0.5
Faster-rcnn	ResNet-50	83.62	38.73	74.89	75.4	0.59
YOLOv3	DarkNet-53	91.37	40.66	84.39	92.33	0.7
YOLOv5S	CSPDarkNet-53	93.21	62.1	85.19	91.44	0.35
Efficientdet-D3	EfficicientNet-B3	86.39	55.67	67.49	89.9	0.77
YOLOv5X	CSPDarkNet-53	98.4	64.8	91.47	99.04	0.69
Centrenet	Hourglass-104	68.51	43.18	84.14	48.74	0.29
Retinanet	ResNet-101	80.79	49.89	66.28	77.56	0.57
YOLOv4	CSPDarkNet-53	96.1	50.06	89.37	94.78	0.56
YOLOR-P6	CSPDarkNet	99.3	59.9	53.5	99	0.63
YOLOX-s	Darknet-53	99.55	70.68	80.32	99.9	0.66
T-model	Our backbone	99.36	69.53	81.1	99.5	0.89
S-model	Our backbone	98.1	65.2	79.44	98.91	0.89

## Data Availability

Aluminium defect dataset can be obtained from https://aistudio.baidu.com/aistudio/datasetdetail/13564 PCB defect dataset can be obtained from https://robotics.pkusz.edu.cn/resources/dataset/PCB/ and NEU surface defect dataset can be obtained from http://faculty.neu.edu.cn/me/songkc/Vision-based_SIS_Steel.html.
